# Spike gene variability in porcine epidemic diarrhea virus as a determinant for virulence

**DOI:** 10.1128/jvi.02165-24

**Published:** 2025-02-26

**Authors:** Wentao Li, Basav N. Hangalapura, Paul van den Elzen, Erwin van den Born, Frank J. M. van Kuppeveld, Peter J. M. Rottier, Berend-Jan Bosch

**Affiliations:** 1 Department of Biomolecular Health Sciences, Division of Infectious Diseases and Immunology, Faculty of Veterinary Medicine, Utrecht University 90051 , Utrecht, the Netherlands; 2 MSD Animal Health 35956 , Boxmeer, the Netherlands; Loyola University Chicago-Health Sciences Campus, Maywood, Illinois, USA

**Keywords:** PEDV, spike, coronavirus, virulence

## Abstract

Porcine epidemic diarrhea virus (PEDV) is a pathogenic coronavirus that targets the swine intestinal tract, leading to acute diarrhea and high mortality in neonatal piglets. PEDV is categorized into different genotypes based on genetic variations, especially in the spike (S) gene. The S protein is crucial for viral entry and a major immune target. Significant differences in virulence have been observed among PEDV genotypes, particularly between classical strains and newly emerging strains. In this study, we explored the impact of spike gene variability on PEDV pathogenicity. Using targeted RNA recombination, we generated recombinant PEDV (rPEDV) variants carrying spike genes from contemporary strains (moderately virulent strain UU and highly virulent strain GDU), all within the genetic background of the avirulent DR13 vaccine strain. Pathogenicity was assessed in 3-day-old piglets. The rPEDV carrying the DR13 spike gene was nonpathogenic, with no detectable viral RNA in feces. The rPEDV with the UU spike gene induced mild to severe diarrhea, with moderate viral shedding but no mortality. Conversely, the rPEDV with the GDU spike gene caused severe diarrhea, high viral titers, and high mortality. These findings highlight the critical role of the spike protein in PEDV virulence, informing future development of effective control strategies, including the design of live-attenuated vaccines.

**IMPORTANCE:**

This study significantly advances our understanding of how genetic variations in the spike (S) protein of porcine epidemic diarrhea virus (PEDV) influence its ability to cause disease. By engineering viruses with spike genes from different PEDV strains, variations in this protein could be directly linked to differences in disease severity. We found that the spike protein from highly virulent strains caused severe diarrhea and high mortality in piglets, while that from less virulent strains led to milder symptoms. These findings emphasize the central role of the spike protein in determining PEDV virulence, which may enable the design of more effective vaccines to combat PEDV and reduce its impact on the swine industry.

## INTRODUCTION

Porcine epidemic diarrhea virus (PEDV) is an enteric coronavirus pathogen in the swine industry, causing significant economic losses, particularly in Asia and North America (1). The emergence of highly virulent PEDV strains in Asia in 2010 and in the USA in 2013 caused widespread outbreaks with near-total mortality in neonatal piglets (2, 3). PEDV strains are categorized into two major genogroups, based on variations in its genome, particularly in the spike (S) gene: genogroup 1 (G1), which includes classical strains such as CV777 and DR13 strains, and genogroup 2 (G2), which includes the US prototype strains. G2 strains are associated with higher virulence and have been linked with more severe outbreaks (1, 4, 5).

The spike protein of PEDV, the major glycoprotein located on the virion surface, is crucial for receptor binding, membrane fusion, and virus entry into host cells, specifically targeting the epithelial cells of the swine intestinal tract (6). As the main antigenic component, the spike is also the prime target for the host’s neutralizing antibody response. Considerable variations in the spike gene have been observed among strains, leading to the “S-Indel” and “non-S-indel” classification. The “S-Indel” strains are clustered within genogroup G1 and G2 and exhibit characteristic insertions and deletions (indels) in the spike (S) gene, which are also present in the classical strains. In contrast, the non-S-indel strains lack these insertions and deletions in the spike gene, fall within the G2 genogroup, and are responsible for severe outbreaks (5).

The differences in pathogenicity across different PEDV strains underscore the need for a better understanding of the viral determinants of virulence, particularly with respect to the suspected role of the S protein in this clinical outcome. Therefore, we generated recombinant PEDVs that carry spike genes from strains with different virulence characteristics and studied their virulence and immunogenicity *in vivo*.

## RESULTS AND DISCUSSION

To evaluate the contribution of the spike protein to PEDV virulence, we employed our previously established reverse genetics system based on the avirulent DR13 vaccine strain (7), which has been widely used as a live-attenuated vaccine in Asia since 2004. This strain, derived from a classical PEDV strain, was attenuated through serial passage in Vero cells (8), resulting in 536 nucleotide mutations and several deletions, many of them located in the spike gene and one 48-nucleotide deletion in ORF3 (9). In contrast to most other PEDV strains, the cell-adapted DR13 showed a trypsin-independent phenotype for propagation in cell culture (6, 10).

**Fig 1:**
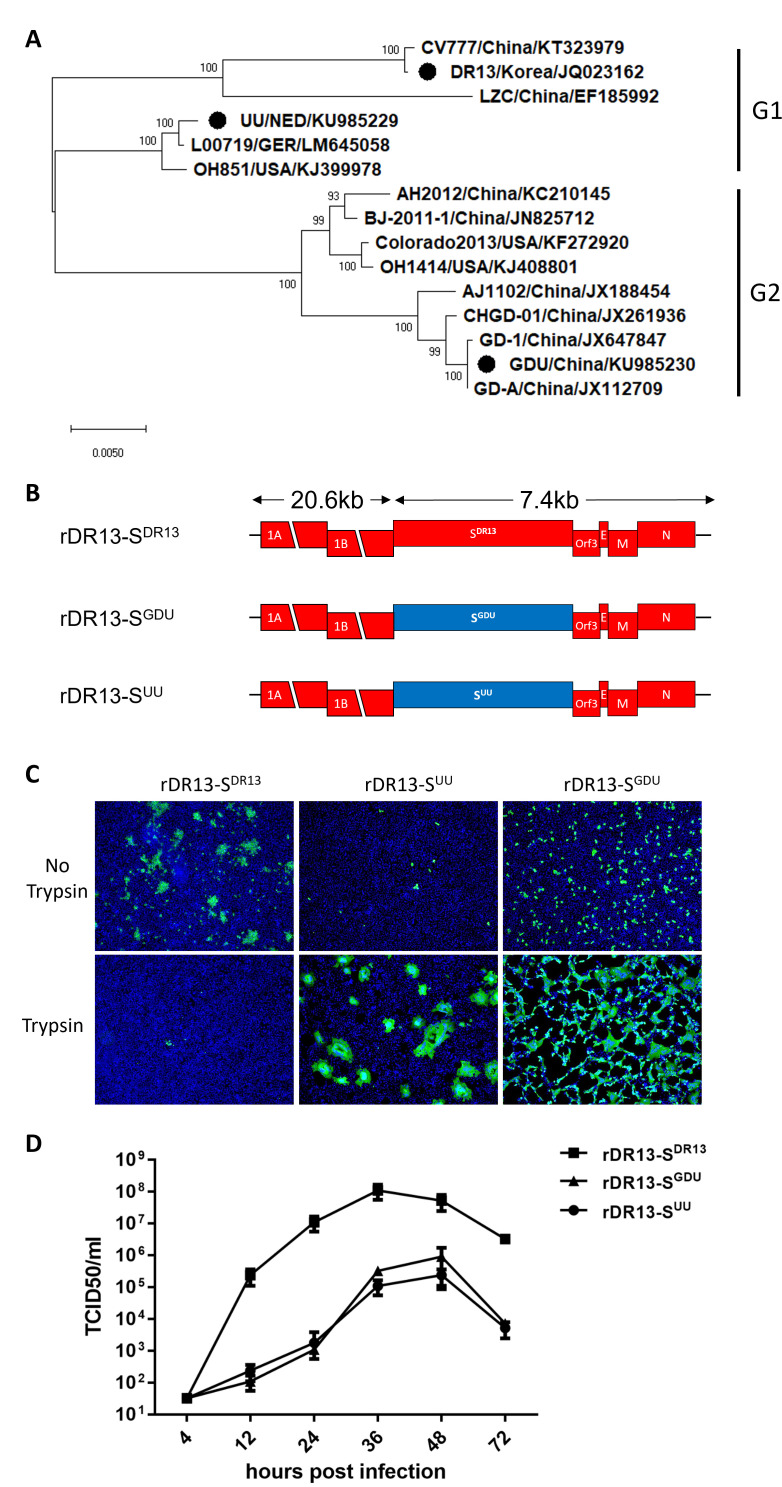
Generation and characterization of recombinant PEDVs. (**A**) Phylogenetic tree of porcine epidemic diarrhea virus (PEDV) generated using spike gene of representative strains, with strains belonging to the G1 and G2 genogroups indicated. The strains used in this study are highlighted by a black dot (•). (**B**) Genomes of recombinant DR13 viruses with the spike gene of the DR13 strain (S^DR13^), the GDU strain (S^GDU^), or the UU strain (S^UU^). (**C**) *In vitro* characterization of recombinant viruses. Vero cells were infected with the indicated viruses at an MOI of 0.1 in the presence (rDR13-S^GDU^ and rDR13-S^UU^) or absence or presence (rDR13-S^DR13^) of 10 µg/mL trypsin. Cells were fixed at 24 hours postinfection and stained with mouse monoclonal antibody 3F12 to the PEDV nucleocapsid protein. Nuclei (blue) were stained with DAPI (blue), and infected cells (green) were visualized by fluorescence microscopy using an anti-PEDV nucleocapsid antibody. (**D**) Multistep growth kinetics of recombinant PEDV viruses. Vero cells were inoculated with each recombinant PEDV (MOI = 0.01) for 3 hours in the presence (rDR13-S^GDU^ and rDR13-S^UU^) or absence (rDR13-S^DR13^) of 10 µg/mL trypsin, after which the inoculum was replaced by fresh culture medium (with or without trypsin). Virus titers were determined at different times postinfection by a quantal assay on Vero cells from which TCID_50_ values were calculated.

We engineered recombinant viruses using the DR13 background and introduced spike genes from two different PEDV strains: the highly virulent non-S-indel G2b strain GDU (GB: KU985230), which shared 99.9% similarity in its spike gene with another highly virulent strain CHGD-01 (11); a mildly virulent Dutch UU strain (S-indel type, GB: KU985229), which is closely related to the US strain OH851 (12); and the original DR13 vaccine strain (GB: JQ023162) (Fig. 1A and B; Fig. S1). Consistent with our previous work (6), infection with the recombinant rDR13-S^UU^ (UU spike gene in DR13 backbone) and rDR13-S^GDU^ (GDU spike gene in DR13 backbone) in Vero cells was enhanced in the presence of trypsin, in contrast to infection with rDR13-S^DR13^ (DR13 spike gene in DR13 backbone) (Fig. 1C). rDR13-S^GDU^ and rDR13-S^UU^ displayed similar replication kinetics, reaching a peak titer of 10^5^ TCID_50_/mL at 48 hours postinfection, while rDR13-S^DR13^ reached a 3-log higher peak titer of 10^8^ TCID_50_/mL at 36 hours postinfection (Fig. 1D).

**Fig 2:**
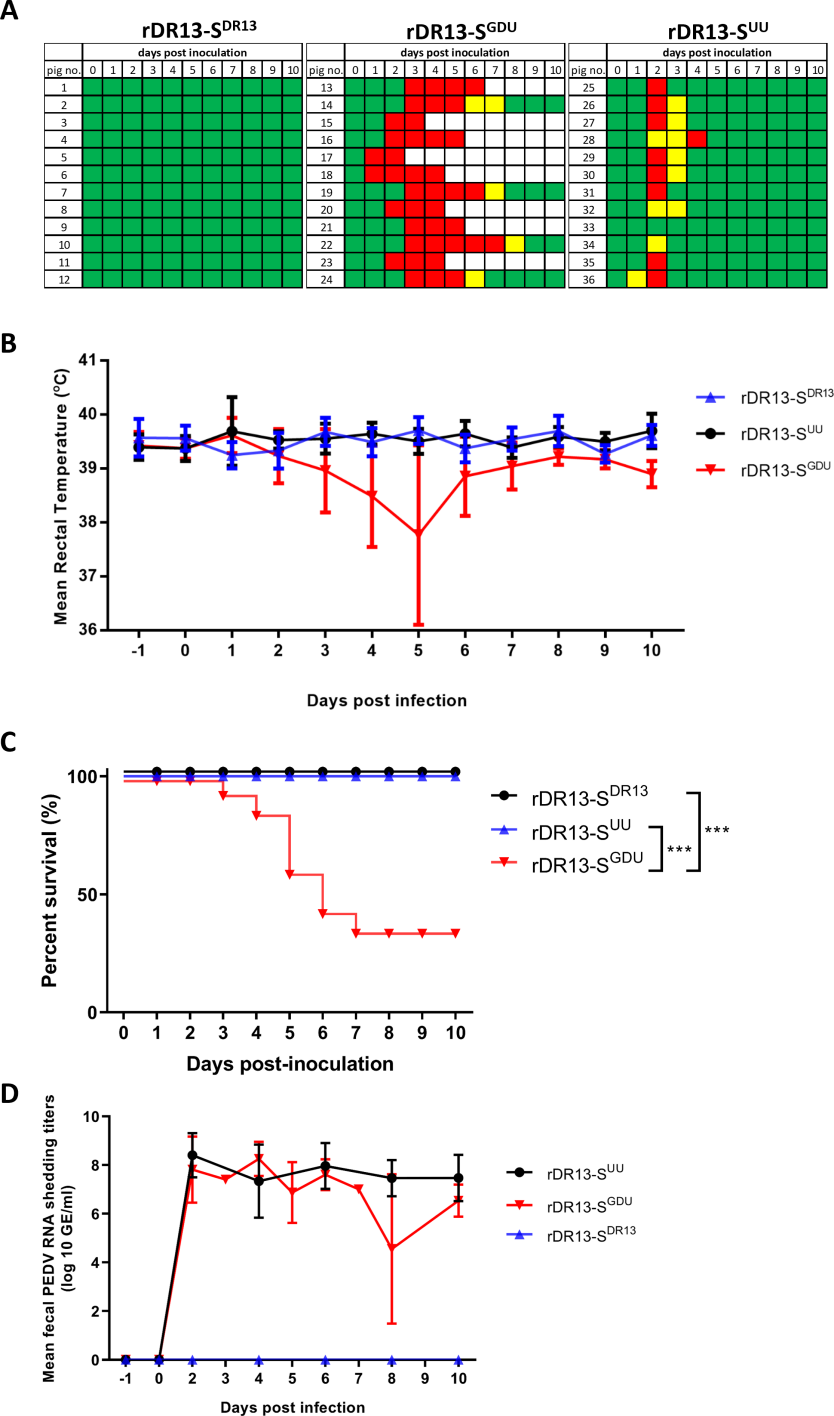
Pathogenicity analysis of recombinant PEDVs in piglets. (**A**) Diarrhea scores of piglets across different groups over time postinoculation. Scoring criteria: green = normal; yellow = mild diarrhea; red = severe diarrhea; white = severe diarrhea requiring euthanasia or resulting in death. (**B**) Average rectal temperatures of piglets infected with the rPEDV variants. (**C**) Kaplan-Meier survival curves displaying the survival rates of piglets infected with the rPEDV variants. The data were analyzed by log-rank test (***, *P* < 0.001). (**D**) Fecal PEDV RNA shedding profiles. Data are displayed as mean values ± standard deviations for each group. Values below the detection limit (1.0 log_10_ GE/mL) were considered negative for viral shedding.

We next evaluated the virulence of these recombinant viruses in approximately 3-day-old piglets. Thirty-six piglets from three litters were divided into three groups (12 piglets per group, cross-fostered by a dam from ~2 days of age) and inoculated orally with 1 × 10^6^ TCID_50_ of each recombinant virus. In the rDR13-S^DR13^ group, none of the piglets showed signs of diarrhea, and no virus shedding was detected in fecal samples (Fig. 2A and D), indicating a complete lack of virulence. In contrast, 91.6% (11/12) of the piglets inoculated with rDR13-S^UU^ developed mild to severe diarrhea at 1–2 days postinfection, but all animals recovered by day 5. The fecal viral shedding titer in this group reached 8.4 ± 0.9 log_10_ GE/mL (genome copies per mL). In the rDR13-S^GDU^ group, all piglets (12/12) developed a severe diarrhea, which was fatal in 66.7% (8/12) of the animals (Fig. 2C). The average fecal viral shedding titer reached 8.3 ± 0.7 log_10_ GE/mL. Piglets in this group also exhibited lower rectal temperatures (Fig. 2B), further confirming the severity of the infection.

**Fig 3:**
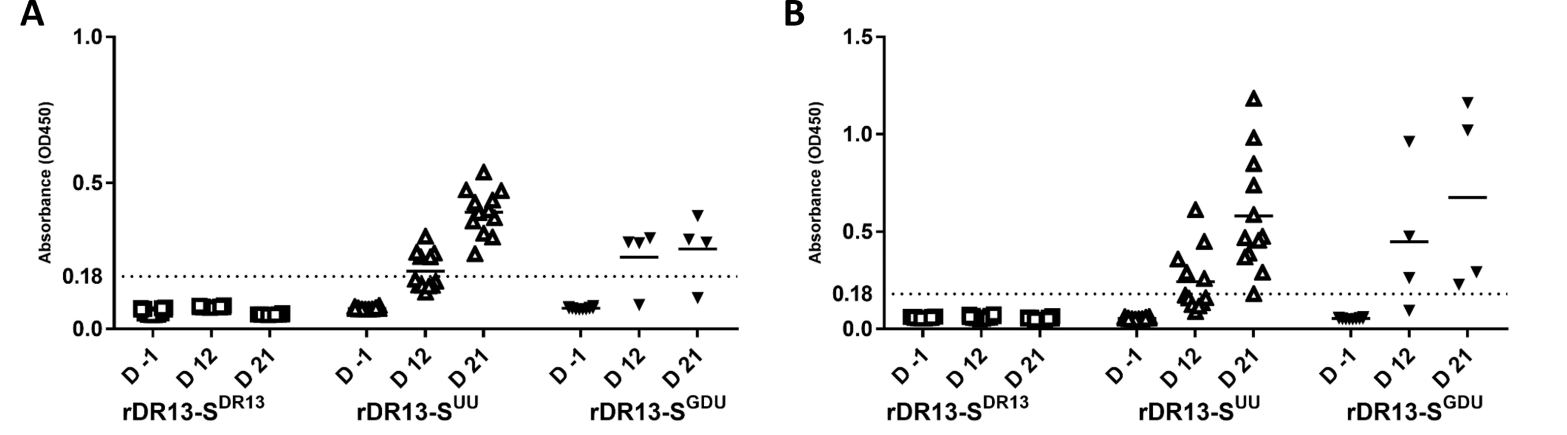
Anti-PEDV-specific IgG and IgA antibody responses following oral inoculation with recombinant PEDVs. The levels of anti-PEDV S1 IgG (**A**) and IgA (**B**) antibodies in serum samples collected at different times pre- or postinfection were quantified by ELISA. D-1 represents 1 day before infection; D 12 and D 21 indicate days 12 and 21 postinfection, respectively.

Serum PEDV-specific IgG (Fig. 3A) and IgA (Fig. 3B) antibodies were measured by PEDV-S1 ELISA. The cutoff value for the S1-ELISA was calculated by using the mean OD value of the serum samples collected 1 day prior to infection, plus three standard deviations (SDs). No seroconversion was observed in the rDR13-S^DR13^ group. In contrast, seroconversion was seen in 50% (6/12) of piglets in the rDR13-S^UU^ group at day 12, with all piglets seroconverting by day 21. Similarly, in the rDR13-S^GDU^ group, seroconversion occurred in 75% (3/4) of the surviving piglets by day 12 and day 21.

This study highlights the critical role of the spike protein in influencing PEDV virulence and immunogenicity. Since the recombinant viruses were generated on the same isogenic background, the observed differences in virulence can be directly attributed to the introduced spike genes. Our data suggest that functional differences in the spike proteins of different PEDV strains, such as receptor binding, interaction with host proteases, and the ability to bind sialic acids, play pivotal roles in virulence. Moreover, we found that restoring the ORF3 gene in the DR13 vaccine strain, which contains a deletion as a result of its adaptation to culturing in Vero cells, was not required for the acquisition of virulence. Our observations align with a recent study by Kristen-Burmann et al., which confirmed that the PEDV spike gene is a significant viral determinant of pathogenicity and further illustrated the modest contribution of an intact ORF3 to viral virulence (13). A similar observation was recently reported for SARS-CoV-2 in a study suggesting that the spike protein is a major virulence determinant for this virus (14).

We previously demonstrated that the spike protein of the GDU strain, but not those of DR13 and UU, binds to sialic acids, a characteristic that correlates with the higher virulence of the GDU strain. We demonstrated that sialic acid binding is crucial for GDU infection in cultured cells and mapped this sialic acid binding activity to the spike protein’s N-terminal domain (GDU strain: a.a. 1–249) (6). Furthermore, PEDV variants lacking this domain exhibited reduced virulence in neonatal piglets (15), supporting the idea that sialic acid binding activity contributes to virus pathogenesis *in vivo*. Other spike protein features, such as affinity for cellular receptors and sensitivity to spike-activating host proteases, may also contribute to PEDV virulence. To further understand the molecular determinants of PEDV virulence, domain-swapping experiments provide a promising avenue for investigation. By exchanging specific regions of the spike protein between strains with differing virulence profiles, such as GDU and DR13, it would be possible to pinpoint which domains or specific sequences contribute to key phenotypic traits including sialic acid binding and sensitivity to receptors and proteases.

Understanding the genetic factors that influence PEDV virulence is crucial for developing safer, more effective vaccines, as elucidating strain-specific virulence mechanisms can better guide strategies to protect swine populations from this devastating virus.

## MATERIALS AND METHODS

### Cells and viruses

Vero-CCL81 (African green monkey kidney cells) were cultured in Dulbecco’s Modified Eagle’s Medium (DMEM, Lonza) supplemented with 10% fetal bovine serum (FBS). For the growth of PEDV, cells were maintained in αMEM-TPB, a medium consisting of Eagle’s minimum essential medium alpha (Life Technologies, 22571–020) supplemented with 0.3% tryptose phosphate broth (TPB, Sigma T9157). Cell culture-adapted PEDV-DR13 (GB: JQ023162), isolated from a GreenCross commercial vaccine from South Korea, along with a recombinant virus encoding the PEDV-DR13 S protein (rDR13-S^DR13^), was propagated and titrated in αMEM-TPB supplemented with 20 mM HEPES in Vero cells (7). Recombinant DR13 strains encoding the spike gene from PEDV field isolates from China (strain GDU, GB: KU985230) and the Netherlands (strain UU, GB: KU985229) were generated as described previously (6, 7, 10) and propagated in αMEM-TPB supplemented with 20 mM HEPES and 10 µg/mL trypsin (Sigma T4799) in Vero cells. Sequencing was used to confirm the replacement of the spike gene in the recombinant viruses.

### Immunofluorescence microscopy

For immunofluorescence staining, cells were rinsed twice with PBS and then fixed with 3.7% formaldehyde (Merck, 1040031000) in PBS. Membranes were permeabilized using 0.1% Triton-X-100 (Sigma, 93426) in PBS for 10 minutes at room temperature. After fixation, the cells were blocked with 3% bovine serum albumin (BSA) (GE Healthcare Life Sciences) in PBS for 1 hour, followed by a 1-hour incubation with mouse anti-PEDV nucleocapsid protein monoclonal antibody (1:200 dilution, 3F12, BioNote, Republic of Korea) in PBS (phosphate-buffered saline) containing 1% BSA. The cells were then rinsed three times with PBST (PBS containing 0.05% Tween-20), and staining was completed using an Alexa Fluor488-conjugated goat anti-mouse antibody (Life Technologies, A11001). DAPI nuclear counterstaining (Molecular Probes) was used to visualize the nuclei. Images of the stained cells were captured at 4 × magnification using an EVOS-FL fluorescence microscope (Thermo Fisher Scientific Inc.).

### Multistep growth analysis

To assess the growth kinetics of the rescued viruses, Vero cells cultured in 24-well plates were infected with the specified viruses at a multiplicity of infection (MOI) of 0.01. After a 3-hour adsorption period, the inoculum was discarded, and the cells were washed twice with PBS (Lonza, USA). The cells were then incubated in αMEM-TPB medium containing 10 µg/mL trypsin (Sigma T4799) at 37°C with 5% CO_2_. At specified times postinfection, both the cell supernatants and infected cells were collected. Virus titers were measured using the endpoint dilution assay and expressed as TCID_50_ per milliliter, following the Reed-Muench method (16).

### Experimental infection in weaned piglets

Thirty-six piglets from three PEDV-naive sows were cross-fostered and randomly assigned to three treatment groups (rDR13-S^DR13^, rDR13-S^UU^, and rDR13-S^GDU^), and each was housed in individual biosafety level 2 (BSL2) rooms. Piglets, aged 3 to 5 days, were orally inoculated with recombinant viruses (rDR13-S^DR13^, rDR13-S^UU^, and rDR13-S^GDU^) at a dose of 1 × 10^6^ TCID_50_. All piglets were kept for the evaluation of morbidity, mortality, and fecal PEDV RNA shedding unless the piglets reached humane endpoint and were euthanized.

Total fecal RNA was extracted using a QIAamp Viral RNA Kits for RNA Extraction (Qiagen, Germany), and the PEDV N gene was detected using the iTaq Universal SYBR Green One-Step RT-qPCR Kit (Bio-Rad; Cat. No. 1725151) using specific primers: PEDV-F4: 5′- AGG GTC GTG GAG CTT CTC AG −3′, PEDV-R4: 5′- CAG CAG CCA CCA GAT CAT CG −3′. All animal experiments were performed according to the protocols approved by the Institutional Animal Care and Use Committee of MSD AH, Boxmeer, the Netherlands.

### Enzyme-linked immunosorbent assay (ELISA)

The pCAGGS mammalian expression vector encoding PEDV S1 (isolate UU, residues 1–725; GB: KU985229), C-terminally extended with the Fc domain of mouse IgG (UU-S1-mFc), was generated as described before (6), and UU-S1-mFc was produced in HEK293T cells as described previously (17). ELISAs were performed as previously described (18). In short, microtiter plates (Greiner bio-one, 655092) were coated with UU-S1-mFc (4.0 ng per well, diluted in PBS) and incubated overnight at 4°C. Following three washes with washing buffer (PBS containing 0.05% Tween 20), plates were blocked with blocking buffer (PBS + 0.1% Tween 20, containing 5% (w/v) skimmed milk) for 3 hours at 37°C and then incubated with sera diluted 1:100 in blocking buffer for 1 hour at 37°C. After washing, a 1:20,000 dilution of horseradish peroxidase (HRP)-conjugated goat anti-pig IgG (Abcam, ab6915) or HRP-conjugated Goat anti-pig IgA (Abcam, ab112746) was added and incubated at 37°C for 1 hour. The peroxidase reaction was visualized using TMB Super Slow One Component HRP Microwell Substrate (BioFX), and optical densities (OD) were measured at 450 nm using the ELISA plate reader (EL-808, Biotek).

### Statistical analysis

Statistical analyses were performed using GraphPad Prism 8.0. The data were analyzed by log-rank test (***, *P* < 0.001).

## Data Availability

All data generated or analyzed during this study are included in this article and its supplementary material files. Genbank accession numbers for sequences used in this study: PEDV DR13 strain (GenBank JQ023162), UU strain (GenBank KU985229) and GDU strain (GenBank KU985230). Additional data sets and raw data supporting the conclusions of this study are available from the corresponding author upon reasonable request.

## SUPPLEMENTAL MATERIAL

Fig. S1 jvi.02165-24-s0001.pdf Sequence alignment of the spike proteins from PEDV strains.

ASM does not own the copyrights to Supplemental Material that may be linked to, or accessed through, an article. The authors have granted ASM a non-exclusive, world-wide license to publish the Supplemental Material files. Please contact the corresponding author directly for reuse.
