# Supplementary material for: Spike gene variability in porcine epidemic diarrhea virus as a determinant for virulence
Source: J Virol. 2025 Feb 26;99(3):e02165-24. doi: 10.1128/jvi.02165-24 (PMC11915861; doi:10.1128/jvi.02165-24)

Supplemental Figure 1

Sequence alignment of the spike proteins from PEDV strains utilized in the study, including DR13 (GenBank: JQ023162) and the UU (GenBank: KU985229) and GDU (GenBank: KU985230).

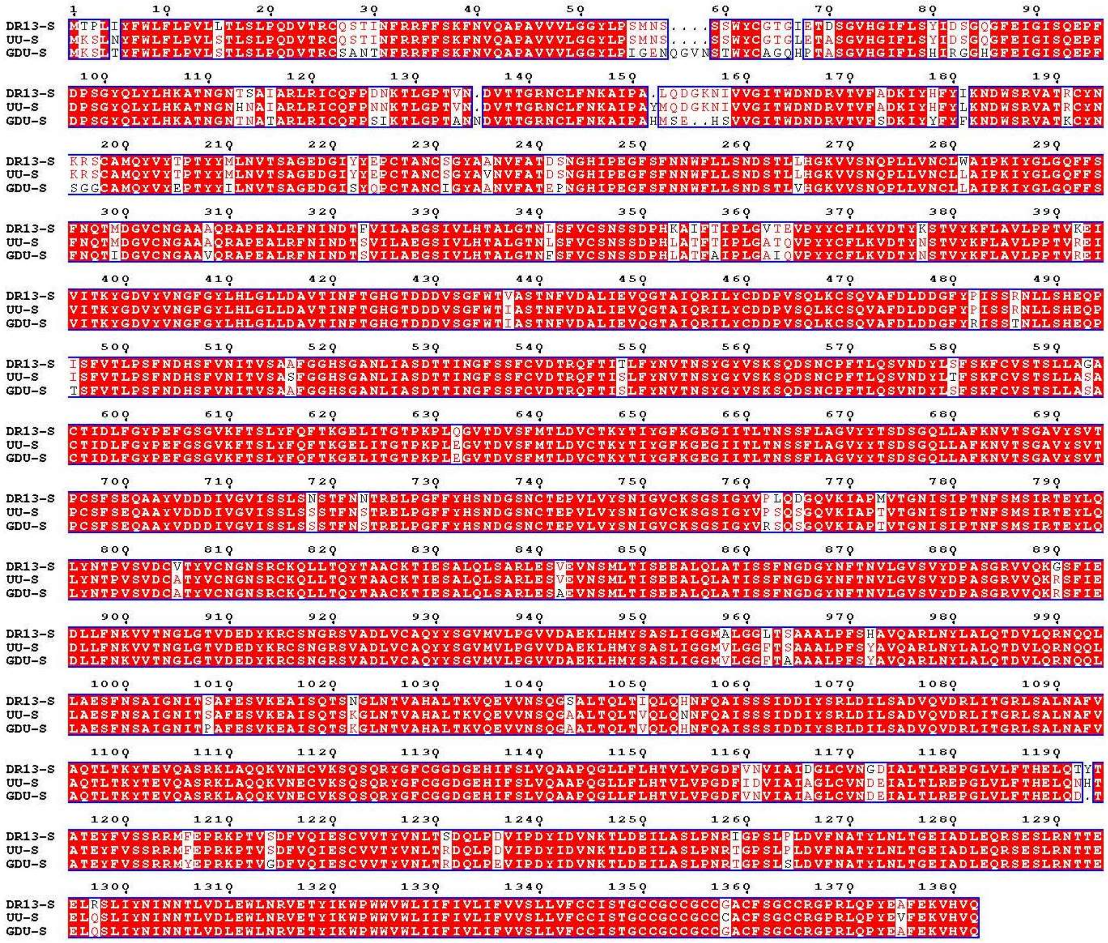

Supplement: Fig. S1 — Sequence alignment of the spike proteins from PEDV strains. [file jvi.02165-24-s0001.pdf]
